# Genetic Risk for Osteoporosis and the Benefit of Adherence to Healthy Lifestyles

**DOI:** 10.3389/ijph.2022.1605114

**Published:** 2022-09-13

**Authors:** Yi-Qun Yang, Xing-Hao Yu, Lin Bo, Shu-Feng Lei, Fei-Yan Deng

**Affiliations:** ^1^ Center for Genetic Epidemiology and Genomics, School of Public Health, Medical College of Soochow University, Suzhou, China; ^2^ Jiangsu Key Laboratory of Preventive and Translational Medicine for Geriatric Diseases, Soochow University, Suzhou, China; ^3^ Department of Rheumatology, The Second Affiliated Hospital of Soochow University, Suzhou, China

**Keywords:** fracture, genetic risk score, osteoporosis, healthy lifestyles, genetic factors

## Abstract

**Objectives:** We aimed to explore how healthy lifestyles and genetic factors influence the risk of Osteoporosis (OP).

**Methods:** In this prospective cohort study, we first performed a genome-wide association study (GWAS) of estimated bone mineral density (eBMD) and constructed the genetic risk score (GRS) based on the effect of single nucleotide polymorphism (SNP) on eBMD. We then assessed the effect of three-level GRS and adherence to healthy lifestyles on the risk of OP and fracture, respectively. Finally, we assessed the joint effects of GRS and lifestyle on the OP and fracture risk.

**Results:** People with higher GRS have a lower risk of OP and fracture. Negative associations were detected between healthy lifestyle factors and the risk of OP and fracture. Compare with the group with high GRS and favorable lifestyles, the group with low GRS and unfavorable lifestyles had a high Hazard Ratio (HR).

**Conclusion:** The findings suggest that adherence to healthy lifestyles can reduce the risk of OP and fracture in people with different genetic risks.

## Introduction

Osteoporosis (OP), known as the invisible killer, is one of the major public health problems, especially in the elderly population. OP is a common systemic skeletal disease characterized by reduced bone mass and altered bone microarchitecture, ultimately leading to skeletal fragility and fractures in different skeletal sites [1, 2]. Bone mineral density (BMD) is an important diagnosis index for OP and a strongly relevant risk factor for fracture as well [3]. BMD is highly heritable, with a heritability of 50%–80% [4–8]. Genome-wide association studies (GWAS) have identified a large number of genetic loci significantly associated with BMD [9, 10]. Besides genetic factors, environmental factors are also important in determining OP risk. The environmental factors include the basic and unchangeable factors (e.g., gender, age, and race) and the changeable lifestyle factors. Lifestyle factors can be divided into two categories: the first category is nutritional factors, such as calcium, protein, cereals, dairy products, fresh fruit and vegetable intake, and the second category is behavioral factors, such as smoking, alcohol consumption, sunshine, and physical activity [11].

Genetic risk score (GRS) is a useful tool in assessing complex genetic diseases by combining multiple genetic loci with small effects and has been widely used in disease risk prediction [12–14]. Recent studies have demonstrated that GRS can predict disease risk more accurately than the rare single gene prediction of disease risk, which offers a wide range of clinical applications in disease control [15–17]. Some previous studies have been conducted to predict the OP risk by calculating the GRS. For example, GRS constructed by using 63 BMD-associated loci was associated with the changes in BMD, while OP is diagnosed on basis of BMD, therefore GRS could be used as a predictor of OP [18]. GRS constructed by multiple genetic loci could improve the predictive accuracy of non-vertebral fracture risk over and above that of clinical risk factors alone, and help stratify individuals by fracture status [19]. However, these studies are limited to genetic factors of disease, environmental factors also play an important role in the development of disease, and the extent to which the incident risk of OP and fracture in individuals with high genetic risk can be offset by a favorable lifestyle remained unclear.

Therefore, based on a large sample of data from UK Biobank, we analyzed the independent and joint effects of genetic and environmental factors on the incidence of OP and fracture risk. First, we performed a new GWAS for eBMD in a subset of the UK Biobank sample. Second, we constructed GRS for eBMD and further analyzed the combined effects of the identified genetic loci from GWAS on OP and fracture risk. Third, we then assessed the relationship between lifestyles and OP risk and classified the individuals according to their lifestyles. Finally, we aimed to assess the joint effect of lifestyle and genetic factors and estimate the extent to which having a favorable lifestyle might reduce the risk of incident OP and fracture in the same cohort, particularly in individuals with high risk scores, as determined by the GRS.

## Methods

### Study Design and Participates

**FIGURE 1:**
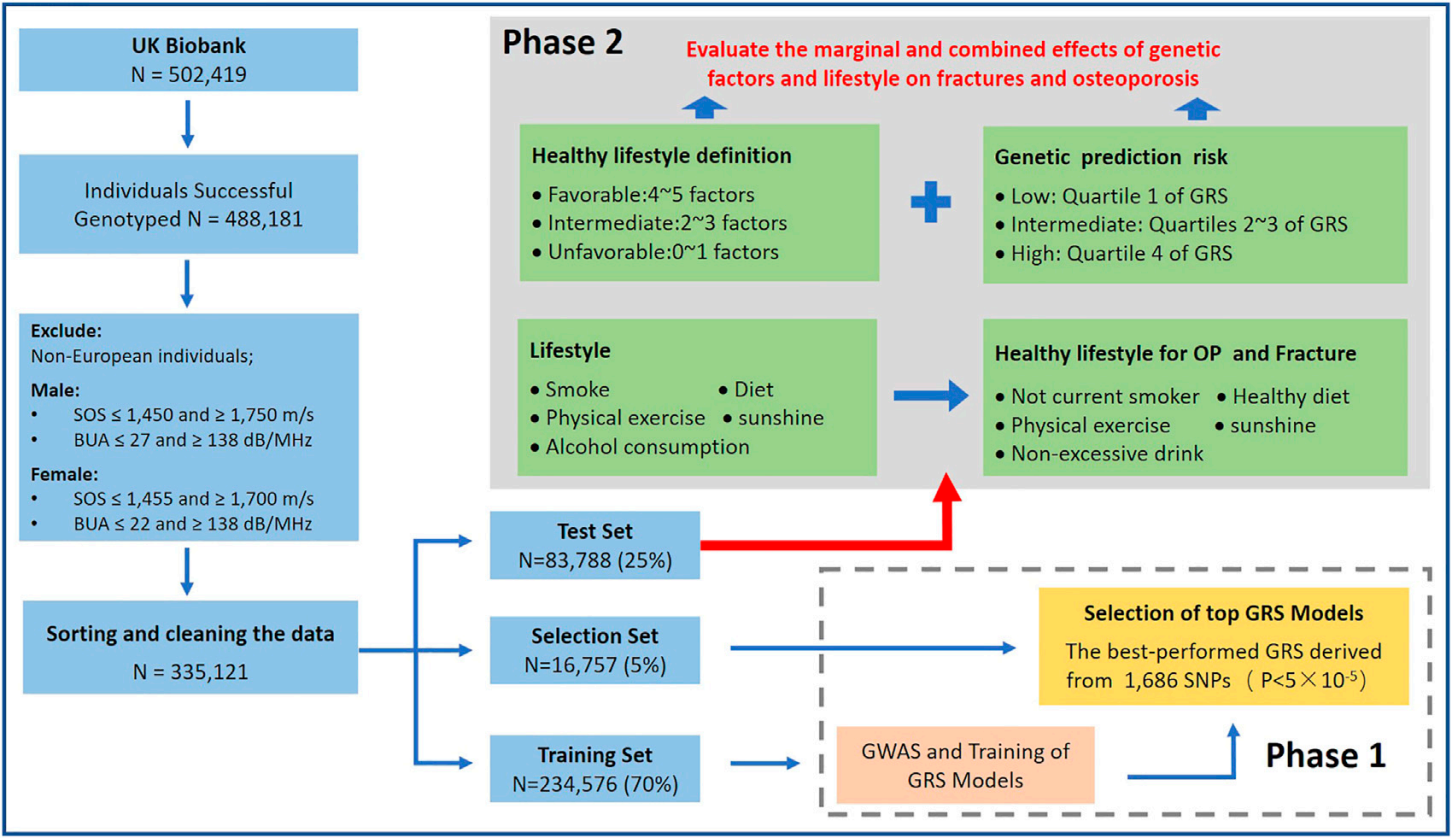
Study design and workflow of our research (Osteoporosis and Fracture, China, 2022).

This study included two parts: 1) Constructing a GRS for estimated bone mineral density (eBMD); 2) Evaluating the separate and joint effects of healthy lifestyles and genetic effects on OP and fracture (Figure 1). To avoid overfitting, we randomly divided whole subjects into training set (70%, *N* = 234,576), selection set (5%, *N* = 16,757) and test set (25%, *N* = 83,788). We first performed a GWAS analysis in the training set and developed five eBMD-based GRSs in both training and test sets under different significance levels. We assessed the strength of the relationship between different GRSs and OP and fracture in the selection set and determined the optimal GRS for further analysis. Finally, in the test set, we evaluated the association between eBMD and various life factors, defined as a healthy lifestyle, and then we explored the separate and combined effects of genetic factors and healthy lifestyles on later OP and fracture risk in the large prospective cohort study.

### UK Biobank Data Sets

As a large-scale biomedical database, UK Biobank contains in-depth genetic and health information from half a million UK participants (http://www.ukbiobank.ac.uk/) [20]. UK Biobank recruited over 500,000 participants aged 40–69 years from 2006 to 2010. The study has collected and continues to collect detailed genotypic and phenotypic information from participants, including questionnaires, physical measurements, multimodal imaging, and for a wide range of health-related longitudinal follow-up investigations [21]. We could obtain detailed health-related information from this project such as biological measurements, lifestyle indicators, biomarkers in blood and urine, etc. This project gives researchers an incredible opportunity to explore the gene-environment joint effects on complex diseases, providing a novel approach to unraveling the process of disease development.

OP and fracture are defined by individual medical records obtained from the International Classification of Diseases Tenth Revision (ICD10) in the UK biobank, which have been widely accepted by previous studies [9, 22, 23] (All the ICD10 codes used are presented in Supplementary Table S1). For the UKB cohort outcomes were defined by the occurrence of OP or fracture, with survival time from baseline to diagnosis, lost to follow-up, death, or by 31 December 2020, whichever came first. All patients who had OP and fractures before entering the cohort were excluded from this study. For individual data, this study retained White British ancestry with detailed and accurate genotype data. We excluded non-European participants (Field ID: 21000) and individuals with unusual large or small eBMD value (eBMD < mean − 4.5SD or eBMD > mean + 4.5SD). The Sahara Clinical Bone Sonometer, which estimates BMD by measuring the speed of sound (SOS) and broadband ultrasound attenuation (BUA) and combining the two to produce a quantitative ultrasound index (QUI). To reduce the impact of outlying measurements, we limit the range of SOS and BUA for the purpose of quality control. The quality control process for the data is detailed in Figure 1.

### Genome-Wide Association Study

We followed a similar quality control by Yu et al. [24] in their osteoporosis study. A total of ∼92 million variants were generated by imputation, which was performed based on Haplotype Reference Consortium (HRC), UK10K and 1,000 Genomes reference panels. We removed SNPs with MAF < 0.01, INFO score < 0.8, Hardy-Weinberg equilibrium *p* < 1 × 10^–7^ and proportion of missingness >0.05. Finally, ∼9,400,000 high-quality SNPs remained for further analysis. We performed a linear mixed model analysis to test the association between autosomal genetic variation and eBMD by using GCTA fastGWA software, assuming an additive allelic effect [25]. The fastGWA is a GWAS analysis tool based on a mixed linear model (MLM) in GCTA software. To explain the genetic structure in the cohort, the sparse genome relationship matrix (GRM) was calculated by individuals of independent European descent from the UK Biobank. This method greatly improves the efficiency of analyzing large data set resources such as the UK Biobank. Similar analyses were also conducted in different gender groups. The following covariates were included as fixed effects: age, gender (Gender covariates were excluded in the gender subgroup analysis), genotyping array, assessment center, and the first 20 principal components. *p* < 5 × 10^–9^ was considered significant in our GWAS analysis. The independence of SNPs was determined with *r* ^2^ < 0.001, which was calculated by using the 1000 Genome Projects reference panel. We identified 1,573 lead SNP signals at genome-wide significance (*p* < 5 × 10^–9^) mapping to 207 loci.

### Healthy Lifestyle Factors

After reviewing extensive literature, we included five healthy lifestyle factors in this research, which are no current smoking [26–28], non-excessive alcohol consumption (moderate drink) [29, 30], physical activity [31–33], healthy diet [34–38], and sunshine exposure [39–42]. “No current smoking” is defined as never having smoked or having quit for at least 30 years. Moderate drink is defined as those who drank once or twice a week or one to three times a month or on special occasions only. Physical activity is defined as ≥150 min of moderate-intensity activity per week, ≥75 min of vigorous exercise per week, at least 5 days of moderate-intensity exercise per week, and one vigorous exercise session per week. A healthy diet is defined as five of the following seven groups, drinking milk (including all types of milk), eating ≥4 servings of fruit per day (Count one apple, one banana, 10 grapes, etc. as one piece; Count one prune, one dried apricot, 10 raisins as one piece), ≥4 servings of vegetables per day (Eating one heaped tablespoon of cooked vegetables or salad or raw vegetables per day as one piece), ≥3 servings of whole grains (Eating 1 bowl of cereal per week as one piece), ≥2 servings of fish (Eating fish more than once a week), ≤1 serving of processed meat and ≤1.5 servings of unprocessed red meat per week. Sunshine is defined as the people who spend ≥2 h outdoor in summer and spend ≥1 h outdoor in winter. The Healthy Lifestyle Index ranges from 0 to 5, with higher scores indicating healthier lifestyles (Detailed information is shown in Supplementary Table S2).

### Construction of Genetic Risk Score

PLINK software was used to combine SNPs into related regions, with the lead SNP for each simply chosen by the highest significance. There were 674, 208, 117, 81, and 55 eBMD-associated SNPs selected within the 1 Mb region at five different levels of significance from 5E-5 to 5E-8, respectively. Then, the lead SNPs generated in the previous step were picked up from UK Biobank individual-level GWAS data sets. The GRS was constructed by-product of the genotypic matrix and effect vector of SNP: $GRS_{i\times 1}=G_{i\times k}\cdot g{\hat{\lambda }}_{k\times 1}$ , where $\hat{\lambda }$ is the *k* vector of estimated effect for *k* lead SNP generated from GWAS analysis, and **G**_*i×k*_ is the matrix of genotypes (0, 1, 2) of *i* individuals and *k* lead SNP.

### Statistical Analysis

Linear regression models were used to assess the relationship between GRS of eBMD and healthy lifestyles after adjusting for confounders [i.e., genotyped batch, assessment center, age, sex, Townsend deprivation index, and top 10 principal components (PCs)]. Multivariable Cox proportional hazards regression models to assess associations between genetic and lifestyle factors and OP/fracture incidence, and hazard ratios (HR) and 95% confidence intervals (CI) were calculated after adjusting for confounders (i.e., genotyped batch, assessment center, age, sex, Townsend deprivation index, and top 10 PCs) to demonstrate the risk of OP/fracture over time in the prospective study. For OP and fractures, we divided them into different subgroups and calculated the cumulative incidence of case in the different subgroups. The proportional hazards (PH) assumption is checked by the cox.zph (Model: The study constructs Cox proportional risk regression model) function in the survival package based on the Schoenfeld residual test. Using Cox regression analysis, we compared the different effects on the risk of developing OP and fracture among people with favorable (adopting four or five healthy lifestyle factors), intermediate (adopting two or three healthy lifestyle factors), and unfavorable (adopting none or one healthy lifestyle factor; Figure 1) lifestyle. A series of sensitivity analyses were conducted to examine the robustness of the results. Trend analysis was performed by the Cochran-Armitage trend test with the “DescTools” package [43]. The fit of the restricted cubic spline (RCS) model is based on the “rms” package and the non-linearity test is based on Wald statistics [44–46]. All statistical analysis for this study was based on R software (version 3.6.1) and PLINK software (version v1.90 b3.38) [47].

## Results

First, we performed a GWAS analysis of eBMD in 70% of the individuals (training set) with both genotype and phenotype data that passed SOS and BUA quality control. Then we randomly divided the remaining 30% of participants into a selection set and test set according to the ratio of 1:5 (Table 1). The selection set was used to screen for the GRS and healthy lifestyle categories that were significantly associated with eBMD, and the other analyses were performed in the test set. In the test set, there are 2,563 OP patients and 4,045 fracture patients accounting for about 3.1% and 4.8% of total subjects, respectively. Table 1 shows the basic characteristics of the studied subjects. In addition, we plotted the frequency density of eBMD in Supplementary Figure S1 for the OP and non-OP, fracture, and non-fracture groups. We included patients with OP and fracture onset, after the baseline date, with a follow-up cutoff date of December 30, 2020, and a median follow-up of 12 years. The flowchart of this study is shown in Figure 1.

**TABLE 1:** Baseline characteristics of the study subjects in the UK Biobank cohort (Osteoporosis and Fracture, China, 2022).

Variable	Osteoporosis (*N* = 83,788)		Fracture (*N* = 83,788)	
	Control	Case	Control	Case
	81,225 (96.9%)	2,563 (3.1%)	79,743 (95.2%)	4,045 (4.8%)
Age (mean (SD))	56.7 (8.00)	61.7 (6.02)	56.7 (8.00)	59.7 (7.39)
Sex (%)				
Men	38,102 (46.9%)	459 (17.9%)	37,165 (46.6%)	1,396 (34.5%)
Women	43,123 (53.1%)	2,104 (82.1%)	42,578 (53.4%)	2,649 (65.5%)
TDI (mean (SD))	−1.60 (2.93)	−1.25 (3.12)	−1.60 (2.92)	−1.31 (3.15)
eBMD (mean (SD))	0.54 (0.13)	0.44 (0.11)	0.54 (0.13)	0.48 (0.12)
eBMD.T (mean (SD))	−0.33 (1.15)	−1.20 (1.05)	−0.33 (1.15)	−0.84 (1.11)
Healthy lifestyle (%)				
No current smoke	48,921 (60.2%)	1,478 (57.7%)	48,086 (60.3%)	2,313 (57.2%)
Physical activity	56,217 (69.2%)	1,605 (62.6%)	55,223 (69.3%)	2,599 (64.3%)
Moderate drink	38,639 (47.6%)	1,268 (49.5%)	37,982 (47.6%)	1,925 (47.6%)
Healthy diet	26,010 (32.0%)	956 (37.3%)	25,619 (32.1%)	1,347 (33.3%)
Sunshine	70,773 (87.1%)	2,195 (85.6%)	69,465 (87.1%)	3,503 (86.6%)
GRS				
High	20,488 (25.2%)	459 (17.9%)	20,158 (25.3%)	789 (19.5%)
Intermediate	40,643 (50.0%)	1,251 (48.8%)	39,936 (50.0%)	1,958 (48.4%)
Low	20,094 (24.7%)	853 (33.3%)	19,649 (24.6%)	1,298 (32.1%)

Notes: TDI, townsend deprivation index; GRS, genetic risk score; eBMD, estimated bone mineral density; eBMD.T, estimated bone mineral density T-score (T-score is the number of standard deviations that the bone density is above or below the standard).

### Selection of Best Performed GRS

**FIGURE 2:**
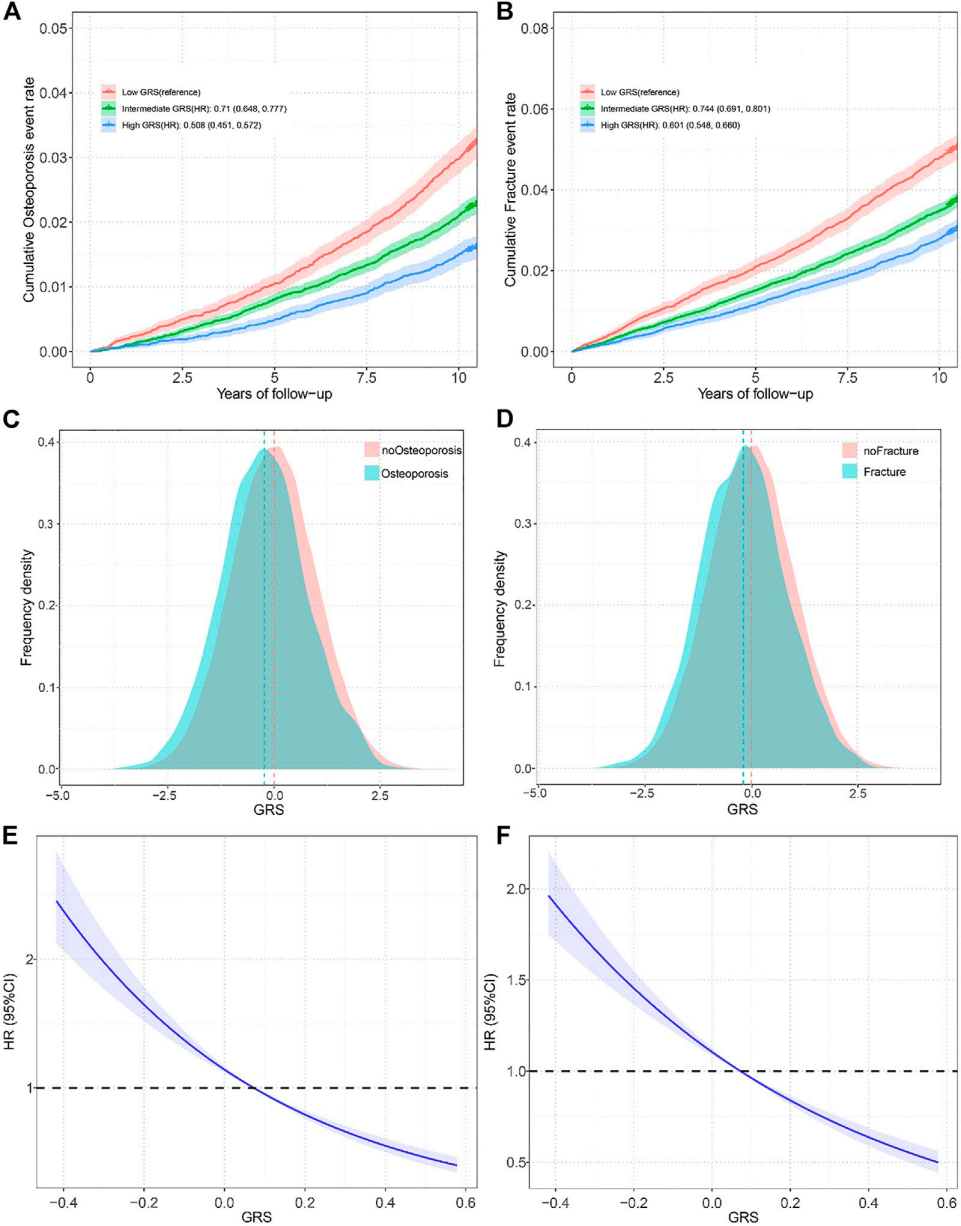
**(A)** Standardized rates of Osteoporosis events in low (bottom quartile), intermediate (quartiles 2–3), and high (top quartile) genetic risk groups in the UK Biobank cohort (Osteoporosis and Fracture, China, 2022); **(B)** Standardized rates of fracture events in low (bottom quartile), intermediate (quartiles 2–3), and high (top quartile) genetic risk groups in the UK Biobank cohort (Osteoporosis and Fracture, China, 2022); **(C)** Distribution of Genetic risk score among participates with and without Osteoporosis in all participates (Osteoporosis and Fracture, China, 2022); **(D)** Distribution of Genetic risk score among participates with and without fracture in all participates (Osteoporosis and Fracture, China, 2022); **(E)** Restricted cubic spline models for the relationship between Genetic risk score with Osteoporosis risk (Osteoporosis and Fracture, China, 2022); **(F)** Restricted cubic spline models for the relationship between Genetic risk score with fracture risk (Osteoporosis and Fracture, China, 2022). Note: Associations were adjusted for age, sex, genotyped batch, assessment center, Townsend deprivation index, and the first 10 principal components of ancestry. OP, osteoporosis: GRS, Genetic risk score; HR, hazard ration.

By using the fast GWAS results from training set, a total of four GRSs for eBMD at different significance levels (*p* < 5 × 10^–5^, *p* < 5 × 10^–6^, *p* < 5 × 10^–7^, *p* < 5 × 10^–8^) were constructed in selection set. We then evaluated the linear relationship between GRS and eBMD in the selection set after adjusting for covariates, and the best-performed GRS for eBMD would be selected to represent genetic components of eBMD for further analysis. The GRS derived from 1,686 SNPs (*p* < 5 × 10^–5^) was proved to have the strongest relationship (*p* = 2.01 × 10^–318^ and beta = 0.037) in the selection set. The associations between the selected GRS and OP/fracture risk in the test set were also most significant (Supplementary Table S3). Meanwhile, compared to patients with OP and fractures, the population usually has a higher GRS (Figures 2C,D).

### The Relationship Between GRS and OP and Fracture

We divided the GRS into low (the bottom quartiles), intermediate (quartiles 2–3), and high (the top quartiles) three levels according to quartiles. After adjusting for covariates, the participants with intermediate GRS (HR = 0.71, 95% CI = 0.648–0.777) and high GRS (HR = 0.508, 95% CI = 0.451–0.572) had a significantly lower risk of OP compared to those with lower GRS (Figure 2A). Gender-stratified analysis showed the same trend except for the men in the intermediate GRS group (Supplementary Table S4). We found similar results for fractures, with an HR of 0.744 (95% CI 0.691–0.801) in the intermediate GRS group and an HR of 0.601 (95% CI 0.548–0.66) in the high GRS group (Figure 2B). The gender-stratified analyses also showed similar associations. A smooth decrease in OP risk with increasing GRS from the first to the tenth decile was found (Supplementary Figure S2A), and similar results were found in the fracture population (Supplementary Figure S2B). In addition, to verify whether there was a non-linear relationship between GRS and OP, we performed trend analysis and plotted restricted cubic spline plots, P_overall_ < 1e-4, P_no-linear_ = 0.3509 results showed that there was no non-linear association, similar results were found in GRS and fracture (P_overall_ < 1e-4, P_non-linear_ = 0.9467).

### Protective Effects of Healthy Lifestyles on OP and Fracture

In the UK Biobank cohort, most participants adopted more than one of the five healthy lifestyle factors: two (19,256 [23.0%] of 83,788 participants), three (28,115 [33.6%] participants) four (19,988 [23.9%] participants) or five (6,092 [7.3%] participants). Based on previous studies (details are shown in the *Methods* section), we finally included five lifestyles in this study that were associated with the occurrence of OP, fracture, or bone mineral density. We demonstrated the significant relationships between these five healthy lifestyles and eBMD by using linear regression after adjusting for covariates (Supplementary Figure S3). For OP, physical exercise was indicated to have a significant effect on the risk reduction with HR estimated to be 0.840 (95% CI = 0.802–0.879, *p* = 4.16E-14); no current smokers had a lower risk of OP compared to smokers (HR = 0.844, 95% CI = 0.807–0.882, *p* = 7.35E-14); and sunshine also showed a protective effect on OP risk (HR = 0.843, 95% CI = 0.782–0.889, *p* = 2.90E-8). However, no significant association was found between moderate alcohol consumption and a healthy diet and the development of OP. For fractures, we only identified protective effects on fracture occurrence of physical activity (HR = 0.871, 95% CI = 0.813–0.932, *p* = 7.43E-5) and no-current smoking (HR = 0.888, 95% CI = 0.831–0.949, *p* = 4.53E-4), while alcohol consumption, diet and Sun exposure have no significant effect. We defined three levels of healthy lifestyle groups based on the eBMD: Favorable group has 4–5 healthy lifestyles (33.8% of OP patients; 32.8% of fracture patients), Intermediate group has 2–3 healthy lifestyles (61.0% of OP patients; 59.4% of fracture patients), Unfavorable has 0–1 healthy lifestyles (5.2% of OP patients; 7.8% of fracture patients). Participants in the unfavorable lifestyle group had a higher relative incidence of OP and fractures than those in the favorable lifestyle group (HR_OP_ = 1.397, 95% CI = 1.204–1.62; HR_fracture_ = 1.385, 95% CI = 1.23–1.56) (Supplementary Table S5).

### Joint Effects of GRS and Healthy Lifestyles on OP and Fracture Risk

**FIGURE 3:**
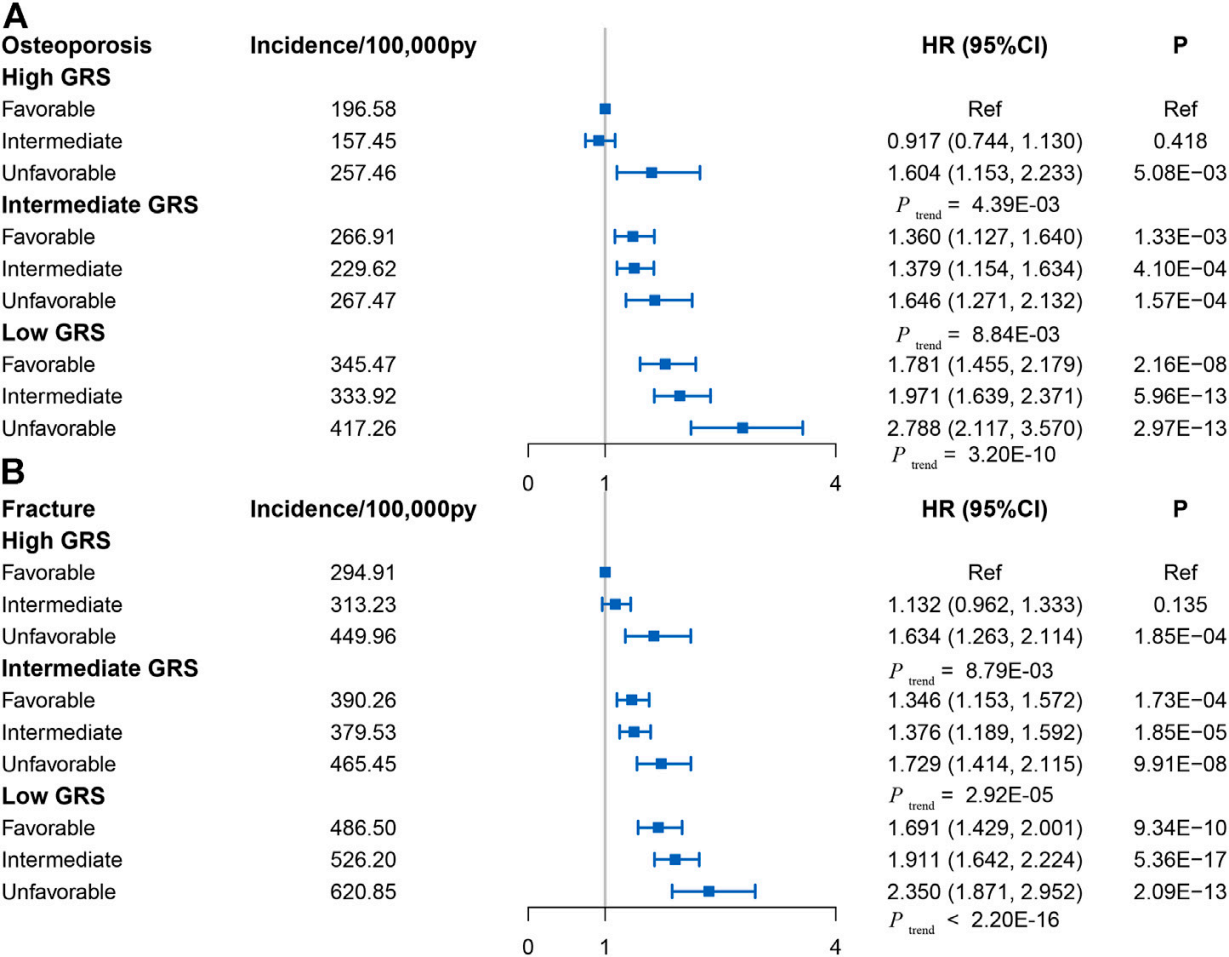
**(A)** Risk of incident osteoporosis in different subgroups defined by Genetic risk score and lifestyle in the UK Biobank cohort (control group was participants with favorable lifestyles in High Genetic risk score group) (Osteoporosis and Fracture, China, 2022). **(B)** Risk of incident fracture in different subgroups defined by Genetic risk score and lifestyle in the UK Biobank cohort (control group was participants with favorable lifestyles in High Genetic risk score group) (Osteoporosis and Fracture, China, 2022). Note: HR, hazard ratio; ref, reference.

Linear regression and Cox analysis were performed to explore the joint effect of GRS and a healthy lifestyle on OP and fracture. First, the linear regression analyses between GRS and lifestyle in the test set indicated no correlation between GRS and lifestyle either overall or single lifestyle (*p* > 0.05), indicating that GRS and lifestyle factors have independent effects in determining OP risk (Supplementary Table S6). Then, Cox regression analyses showed a joint effect of GRS and lifestyle factors on the dose-response manner of the risk of incident OP and fracture, with the risk of disease increasing as GRS decreases and healthy lifestyle factors reduce (Figures 3A,B; Supplementary Figure S4). Trend tests were conducted and all *p*-values were less than 0.05 as detailed in Figure 3.

## Discussion

Genetic factors and a healthy lifestyle act in concert to influence the incidence of OP and fracture risk. In this study, we conducted a GWAS analysis of eBMD based on the UK Biobank cohort and constructed a series of GRSs to assess the effect of genetic risk on overall incident OP and fracture risk in this cohort. The results of this study suggest that a low genetic risk (High GRS) is associated with a reduced risk of OP and fracture independently of lifestyle factors in the large community-based population. Participants with low GRS and an unfavorable lifestyle had the greatest incidence of OP and fracture risk compared to those with high GRS and a favorable healthy lifestyle. Within and across genetic risk groups, adherence to favorable lifestyle behaviors was associated with a reduced incidence of OP and fracture. To our knowledge, this study is the first to systematically assess the risk of OP and fracture by combining genetic risk and lifestyle factors.

Smoking has been identified as one of the explicit risk factors for OP [48], and the results of this study also support this view. A recent study has shown that carcinogens in cigarettes can bind to the aryl hydrocarbon receptors (AHRS) in human cells, stimulating osteoclast abnormalities and increasing bone resorption by activating the cytochrome P450 enzyme [49]. Early tobacco consumption was associated with low bone mineral density in late adolescence [50, 51]. In young women, a dose-response decrease in BMD was associated with increased tobacco consumption [52]. In an older population, meta-analysis has shown that cigarette smoking is associated with reduced bone mass, increased bone loss [53], and a higher risk of fracture [54]. Meanwhile, quitting smoking has been clearly shown to improve BMD and reduce the risk of fracture [55].

Our findings suggest that moderate physical activity is inversely associated with the occurrence of OP and fractures. The commonly accepted view is that a mechanical loading above the daily activity is required to provide BMD. Therefore, appropriate physical activity or exercise has a positive effect on bone mass throughout life. Physical activity or exercise plays a positive role in the development of healthy bones, both during childhood and adolescence [56]. High impact loading improves bone health in premenopausal women, according to an 18-month RCT [57]. Another RCT study also assessed the effects of high-intensity progressive resistance training (HiPRT) on bone health. Their results showed improvements in BMD in both the lumbar spine and femoral neck compared to the control group [58].

The principal route of the effect of sunshine exposure on bone health may be to influence the endogenous synthesis of vitamin D in the body. Elevated serum 25-hydroxyvitamin D (25[OH]D) increases intestinal absorption of calcium and phosphorus by reducing parathyroid hormone secretion (PTH), ultimately increasing BMD [59, 60]. On the other hand, a rat experiment showed that sunshine exposure was positively associated with an improved bone structure [41]. In another study, the authors stratified the cumulative sunshine exposure of the survey population, decreasing the prevalence of fractures in women as the levels increased [40]. Although there is growing evidence that sunshine exposure has a positive effect on bone health, clear mechanisms of action still need to be explored further.

The main strengths of our study are the large sample size and the cohort design with long-term follow-up. The large sample size enables us to find risk factors with relatedly small effects, while the results of our study confirm a direction of further research, and provide strong evidence to support an association between adherence to a favorable lifestyle and a reduced incidence of OP and fracture risk. People with a high genetic risk (Low GRS) have a higher risk of OP and fracture, and this finding helps us to identify individuals at high risk. At the same time, our study shows that adherence to a healthy lifestyle can reduce the risk of OP and fracture both within and across genetic risk groups.

There are several limitations to our study. First, to avoid the interference of race, participants in this study are all of the European ancestry and therefore the findings are limited in their generalization to other ancestries. Second, lifestyle is a dynamic process, but the lifestyle factors covered in this study were obtained from baseline data, and changes in behaviors during follow-up might have affected risk estimates. Third, only lead SNPs were used to de-construct the GRS and therefore may lead to a lack of polygenic effects, and further studies are needed to compare the predictive power of polygenic risk scores with GRS. Fourth, previous studies have shown that the use of ICD10 in UK Biobank to diagnose disease may be affected by bias (e.g., pre-existing disease, drug use). Fortunately, this adverse effect may be offset by large sample sizes in cohort studies. And ICD10 has higher accuracy than identifying disease through patient self-reporting.

### Conclusion

In conclusion, our study shows that both genetic and lifestyle factors have a significant effect on the risk of OP and fracture incidence. Among individuals at high genetic risk, those with a favorable life have a lower risk of OP or fracture compared to those with an unfavorable life. These findings suggest that people who are genetically susceptible to OP or fracture can reduce their risk of OP or fracture by adhering to healthier lifestyles.

## Data Availability

The UK Biobank data were obtained through direct application to the UK Biobank (ID: 76875) and can be downloaded from https://www.ukbiobank.ac.uk/.

## References

[1] NIH Consensus Development Panel on Osteoporosis Prevention, Diagnosis, and Therapy. Osteoporosis Prevention, Diagnosis, and Therapy. JAMA (2001) 285(6):785–95. 10.1001/jama.285.6.785 11176917

[2] Ensrud KE Crandall CJ. Osteoporosis. Ann Intern Med (2017) 167(3):ITC17–ITC32. 10.7326/AITC201708010 28761958

[3] Johnell O Kanis JA Oden A Johansson H De Laet C Delmas P Predictive Value of BMD for Hip and Other Fractures. J Bone Miner Res (2005) 20(7):1185–94. 10.1359/JBMR.050304 15940371

[4] Arden NK Baker J Hogg C Baan K Spector TD. The Heritability of Bone mineral Density, Ultrasound of the Calcaneus and Hip axis Length: a Study of Postmenopausal Twins. J Bone Miner Res (1996) 11(4):530–4. 10.1002/jbmr.5650110414 8992884

[5] Hunter DJ de Lange M Andrew T Snieder H MacGregor AJ Spector TD. Genetic Variation in Bone mineral Density and Calcaneal Ultrasound: a Study of the Influence of Menopause Using Female Twins. Osteoporos Int (2001) 12(5):406–11. 10.1007/s001980170110 11444090

[6] Bauer DC Glüer CC Cauley JA Vogt TM Ensrud KE Genant HK Broadband Ultrasound Attenuation Predicts Fractures Strongly and Independently of Densitometry in Older Women. A Prospective Study. Study of Osteoporotic Fractures Research Group. Arch Intern Med (1997) 157(6):629–34. 10.1001/archinte.157.6.629 9080917

[7] Bauer DC Ewing SK Cauley JA Ensrud KE Cummings SR Orwoll ES Quantitative Ultrasound Predicts Hip and Non-spine Fracture in Men: the MrOS Study. Osteoporos Int (2007) 18(6):771–7. 10.1007/s00198-006-0317-5 17273893

[8] Karasik D Myers RH Hannan MT Gagnon D McLean RR Cupples LA Mapping of Quantitative Ultrasound of the Calcaneus Bone to Chromosome 1 by Genome-wide Linkage Analysis. Osteoporos Int (2002) 13(10):796–802. 10.1007/s001980200110 12378368

[9] Morris JA Kemp JP Youlten SE Laurent L Logan JG Chai RC An Atlas of Genetic Influences on Osteoporosis in Humans and Mice. Nat Genet (2019) 51(2):258–66. 10.1038/s41588-018-0302-x 30598549 PMC6358485

[10] Richards JB Zheng H-F Spector TD. Genetics of Osteoporosis from Genome-wide Association Studies: Advances and Challenges. Nat Rev Genet (2012) 13(8):576–88. 10.1038/nrg3228 22805710

[11] Zhu K Prince RL. Lifestyle and Osteoporosis. Curr Osteoporos Rep (2015) 13(1):52–9. 10.1007/s11914-014-0248-6 25416958

[12] Dai J Lv J Zhu M Wang Y Qin N Ma H Identification of Risk Loci and a Polygenic Risk Score for Lung Cancer: a Large-Scale Prospective Cohort Study in Chinese Populations. Lancet Respir Med (2019) 7(10):881–91. 10.1016/S2213-2600(19)30144-4 31326317 PMC7015703

[13] Natarajan P Young R Stitziel NO Padmanabhan S Baber U Mehran R Polygenic Risk Score Identifies Subgroup with Higher Burden of Atherosclerosis and Greater Relative Benefit from Statin Therapy in the Primary Prevention Setting. Circulation (2017) 135(22):2091–101. 10.1161/CIRCULATIONAHA.116.024436 28223407 PMC5484076

[14] Trajanoska K Morris JA Oei L Zheng H-F Evans DM Kiel DP Assessment of the Genetic and Clinical Determinants of Fracture Risk: Genome Wide Association and Mendelian Randomisation Study. BMJ (2018) 362:k3225. 10.1136/bmj.k3225 30158200 PMC6113773

[15] Khera AV Chaffin M Aragam KG Haas ME Roselli C Choi SH Genome-wide Polygenic Scores for Common Diseases Identify Individuals with Risk Equivalent to Monogenic Mutations. Nat Genet (2018) 50(9):1219–24. 10.1038/s41588-018-0183-z 30104762 PMC6128408

[16] Khoury MJ Armstrong GL Bunnell RE Cyril J Iademarco MF. The Intersection of Genomics and Big Data with Public Health: Opportunities for Precision Public Health. Plos Med (2020) 17(10):e1003373. 10.1371/journal.pmed.1003373 33119581 PMC7595300

[17] Lewis CM Vassos E. Polygenic Risk Scores: from Research Tools to Clinical Instruments. Genome Med (2020) 12(1):44. 10.1186/s13073-020-00742-5 32423490 PMC7236300

[18] Mitchell JA Chesi A Elci O McCormack SE Roy SM Kalkwarf HJ Genetic Risk Scores Implicated in Adult Bone Fragility Associate with Pediatric Bone Density. J Bone Miner Res (2016) 31(4):789–95. 10.1002/jbmr.2744 26572781 PMC4826827

[19] Ho-Le TP Center JR Eisman JA Nguyen HT Nguyen TV. Prediction of Bone Mineral Density and Fragility Fracture by Genetic Profiling. J Bone Miner Res (2017) 32(2):285–93. 10.1002/jbmr.2998 27649491

[20] Rusk N. The UK Biobank. Nat Methods (2018) 15(12):1001. 10.1038/s41592-018-0245-2 30504882

[21] Sudlow C Gallacher J Allen N Beral V Burton P Danesh J UK Biobank: an Open Access Resource for Identifying the Causes of a Wide Range of Complex Diseases of Middle and Old Age. Plos Med (2015) 12(3):e1001779. 10.1371/journal.pmed.1001779 25826379 PMC4380465

[22] Forgetta V Keller-Baruch J Forest M Durand A Bhatnagar S Kemp JP Development of a Polygenic Risk Score to Improve Screening for Fracture Risk: A Genetic Risk Prediction Study. Plos Med (2020) 17(7):e1003152. 10.1371/journal.pmed.1003152 32614825 PMC7331983

[23] Lu T Forgetta V Keller-Baruch J Nethander M Bennett D Forest M Improved Prediction of Fracture Risk Leveraging a Genome-wide Polygenic Risk Score. Genome Med (2021) 13(1):16. 10.1186/s13073-021-00838-6 33536041 PMC7860212

[24] Yu XH Wei YY Zeng P Lei SF. Birth Weight Is Positively Associated with Adult Osteoporosis Risk: Observational and Mendelian Randomization Studies. J bone mineral Res (2021) 36:1469–80. 10.1002/jbmr.4316 34105796

[25] Jiang L Zheng Z Qi T Kemper KE Wray NR Visscher PM A Resource-Efficient Tool for Mixed Model Association Analysis of Large-Scale Data. Nat Genet (2019) 51(12):1749–55. 10.1038/s41588-019-0530-8 31768069

[26] Cusano NE. Skeletal Effects of Smoking. Curr Osteoporos Rep (2015) 13(5):302–9. 10.1007/s11914-015-0278-8 26205852

[27] Wong PKK Christie JJ Wark JD. The Effects of Smoking on Bone Health. Clin Sci (2007) 113(5):233–41. 10.1042/CS20060173 17663660

[28] Law MR Hackshaw AK. A Meta-Analysis of Cigarette Smoking, Bone mineral Density and Risk of Hip Fracture: Recognition of a Major Effect. BMJ (1997) 315(7112):841–6. 10.1136/bmj.315.7112.841 9353503 PMC2127590

[29] Sampson HW. Alcohol and Other Factors Affecting Osteoporosis Risk in Women. Alcohol Res Health (2002) 26(4):292–8. 12875040 PMC6676684

[30] Felson DT Zhang Y Hannan MT Kannel WB Kiel DP. Alcohol Intake and Bone mineral Density in Elderly Men and Women. The Framingham Study. Am J Epidemiol (1995) 142(5):485–92. 10.1093/oxfordjournals.aje.a117664 7677127

[31] Baxter-Jones ADG Kontulainen SA Faulkner RA Bailey DA. A Longitudinal Study of the Relationship of Physical Activity to Bone mineral Accrual from Adolescence to Young Adulthood. Bone (2008) 43(6):1101–7. 10.1016/j.bone.2008.07.245 18725335

[32] Nilsson M Ohlsson C Mellström D Lorentzon M. Previous Sport Activity during Childhood and Adolescence Is Associated with Increased Cortical Bone Size in Young Adult Men. J Bone Miner Res (2009) 24(1):125–33. 10.1359/jbmr.080909 18767931

[33] Michaëlsson K Olofsson H Jensevik K Larsson S Mallmin H Berglund L Leisure Physical Activity and the Risk of Fracture in Men. Plos Med (2007) 4(6):e199. 10.1371/journal.pmed.0040199 17579509 PMC1892039

[34] Levis S Lagari VS. The Role of Diet in Osteoporosis Prevention and Management. Curr Osteoporos Rep (2012) 10(4):296–302. 10.1007/s11914-012-0119-y 23001895

[35] Hamidi M Boucher BA Cheung AM Beyene J Shah PS. Fruit and Vegetable Intake and Bone Health in Women Aged 45 Years and over: a Systematic Review. Osteoporos Int (2011) 22(6):1681–93. 10.1007/s00198-010-1510-0 21165601

[36] Choi E Park Y. The Association between the Consumption of Fish/Shellfish and the Risk of Osteoporosis in Men and Postmenopausal Women Aged 50 Years or Older. Nutrients (2016) 8(3):113. 10.3390/nu8030113 26927165 PMC4808843

[37] Mangano KM Sahni S Kiel DP Tucker KL Dufour AB Hannan MT. Bone Mineral Density and Protein-Derived Food Clusters from the Framingham Offspring Study. J Acad Nutr Diet (2015) 115(10):1605–13. 10.1016/j.jand.2015.04.001 26038297 PMC4584170

[38] Hayhoe RP Lentjes MA Luben RN Khaw KT Welch AA. Dietary Magnesium and Potassium Intakes and Circulating Magnesium Are Associated with Heel Bone Ultrasound Attenuation and Osteoporotic Fracture Risk in the EPIC-Norfolk Cohort Study. Am J Clin Nutr (2015) 102(2):376–84. 10.3945/ajcn.114.102723 26135346

[39] Brouwer-Brolsma EM Vaes AMM van der Zwaluw NL van Wijngaarden JP Swart KMA Ham AC Relative Importance of Summer Sun Exposure, Vitamin D Intake, and Genes to Vitamin D Status in Dutch Older Adults: The B-PROOF Study. J Steroid Biochem Mol Biol (2016) 164:168–76. 10.1016/j.jsbmb.2015.08.008 26275945

[40] Thompson MJW Aitken DA Otahal P Cicolini J Winzenberg TM Jones G. The Relationship between Cumulative Lifetime Ultraviolet Radiation Exposure, Bone mineral Density, Falls Risk and Fractures in Older Adults. Osteoporos Int (2017) 28(7):2061–8. 10.1007/s00198-017-4001-8 28321507

[41] Abulmeaty MMA. Sunlight Exposure vs. Vitamin D Supplementation on Bone Homeostasis of Vitamin D Deficient Rats. Clin Nutr Exp (2017) 11:1–9. 10.1016/j.yclnex.2016.10.003

[42] Min CY Yoo DM Choi HG. Associations between Physical Activity, Sunshine Duration and Osteoporosis According to Obesity and Other Lifestyle Factors: A Nested Case-Control Study. Int J Environ Res Public Health (2021) 18(9):4437. 10.3390/ijerph18094437 33922027 PMC8122401

[43] Agresti A. Categorical Data Analysis. Hoboken, New Jersey, US: John Wiley & Sons (2003).

[44] Smith PL. Splines as a Useful and Convenient Statistical Tool. The Am Statistician (1979) 33(2):57–62. 10.2307/2683222

[45] Govindarajulu US Spiegelman D Thurston SW Ganguli B Eisen EA. Comparing Smoothing Techniques in Cox Models for Exposure-Response Relationships. Stat Med (2007) 26(20):3735–52. 10.1002/sim.2848 17538974

[46] Durrleman S Simon R. Flexible Regression Models with Cubic Splines. Stat Med (1989) 8(5):551–61. 10.1002/sim.4780080504 2657958

[47] Chambers JM. Software for Data Analysis: Programming with R. Berlin, Germany: Springer (2008).

[48] Kelsey JL, Risk Factors for Osteoporosis and Associated Fractures. Public Health Rep (1989) 104:14–20. PMC1580372 2517695

[49] Iqbal J Sun L Cao J Yuen T Lu P Bab I Smoke Carcinogens Cause Bone Loss through the Aryl Hydrocarbon Receptor and Induction of Cyp1 Enzymes. Proc Natl Acad Sci U S A (2013) 110(27):11115–20. 10.1073/pnas.1220919110 23776235 PMC3704019

[50] Eleftheriou KI Rawal JS James LE Payne JR Loosemore M Pennell DJ Bone Structure and Geometry in Young Men: the Influence of Smoking, Alcohol Intake and Physical Activity. Bone (2013) 52(1):17–26. 10.1016/j.bone.2012.09.003 22985892

[51] Lucas R Fraga S Ramos E Barros H. Early Initiation of Smoking and Alcohol Drinking as a Predictor of Lower Forearm Bone mineral Density in Late Adolescence: a Cohort Study in Girls. PloS one (2012) 7(10):e46940. 10.1371/journal.pone.0046940 23094033 PMC3475705

[52] Callréus M McGuigan F Akesson K. Adverse Effects of Smoking on Peak Bone Mass May Be Attenuated by Higher Body Mass index in Young Female Smokers. Calcif Tissue Int (2013) 93(6):517–25. 10.1007/s00223-013-9785-8 24005807

[53] Ward KD Klesges RC. A Meta-Analysis of the Effects of Cigarette Smoking on Bone mineral Density. Calcif Tissue Int (2001) 68(5):259–70. 10.1007/BF02390832 11683532 PMC5352985

[54] Kanis JA Johnell O Oden A Johansson H De Laet C Eisman JA Smoking and Fracture Risk: a Meta-Analysis. Osteoporos Int (2005) 16(2):155–62. 10.1007/s00198-004-1640-3 15175845

[55] Oncken C Prestwood K Kleppinger A Wang Y Cooney J Raisz L. Impact of Smoking Cessation on Bone mineral Density in Postmenopausal Women. J Womens Health (2006) 15(10):1141–50. 10.1089/jwh.2006.15.1141 17199455

[56] Daly RM. The Effect of Exercise on Bone Mass and Structural Geometry during Growth. Med Sport Sci (2007) 51:33–49. 10.1159/000103003 17505118

[57] Heinonen A Kannus P Sievänen H Oja P Pasanen M Rinne M Randomised Controlled Trial of Effect of High-Impact Exercise on Selected Risk Factors for Osteoporotic Fractures. Lancet (London, England) (1996) 348(9038):1343–7. 10.1016/S0140-6736(96)04214-6 8918277

[58] Watson SL Weeks BK Weis LJ Horan SA Beck BR. Heavy Resistance Training Is Safe and Improves Bone, Function, and Stature in Postmenopausal Women with Low to Very Low Bone Mass: Novel Early Findings from the LIFTMOR Trial. Osteoporos Int (2015) 26(12):2889–94. 10.1007/s00198-015-3263-2 26243363

[59] Audran M Kumar R. The Physiology and Pathophysiology of Vitamin D. Mayo Clin Proc (1985) 60(12):851–66. 10.1016/s0025-6196(12)64791-0 3906291

[60] Todd JJ Pourshahidi LK McSorley EM Madigan SM Magee PJ. Vitamin D: Recent Advances and Implications for Athletes. Sports Med (2015) 45(2):213–29. 10.1007/s40279-014-0266-7 25252613

