# Supplementary material for: Genetic Risk for Osteoporosis and the Benefit of Adherence to Healthy Lifestyles
Source: Int J Public Health. 2022 Sep 13;67:1605114. doi: 10.3389/ijph.2022.1605114 (PMC9513025; doi:10.3389/ijph.2022.1605114)
Supplement: Supplementary file 1 [file DataSheet1.doc]

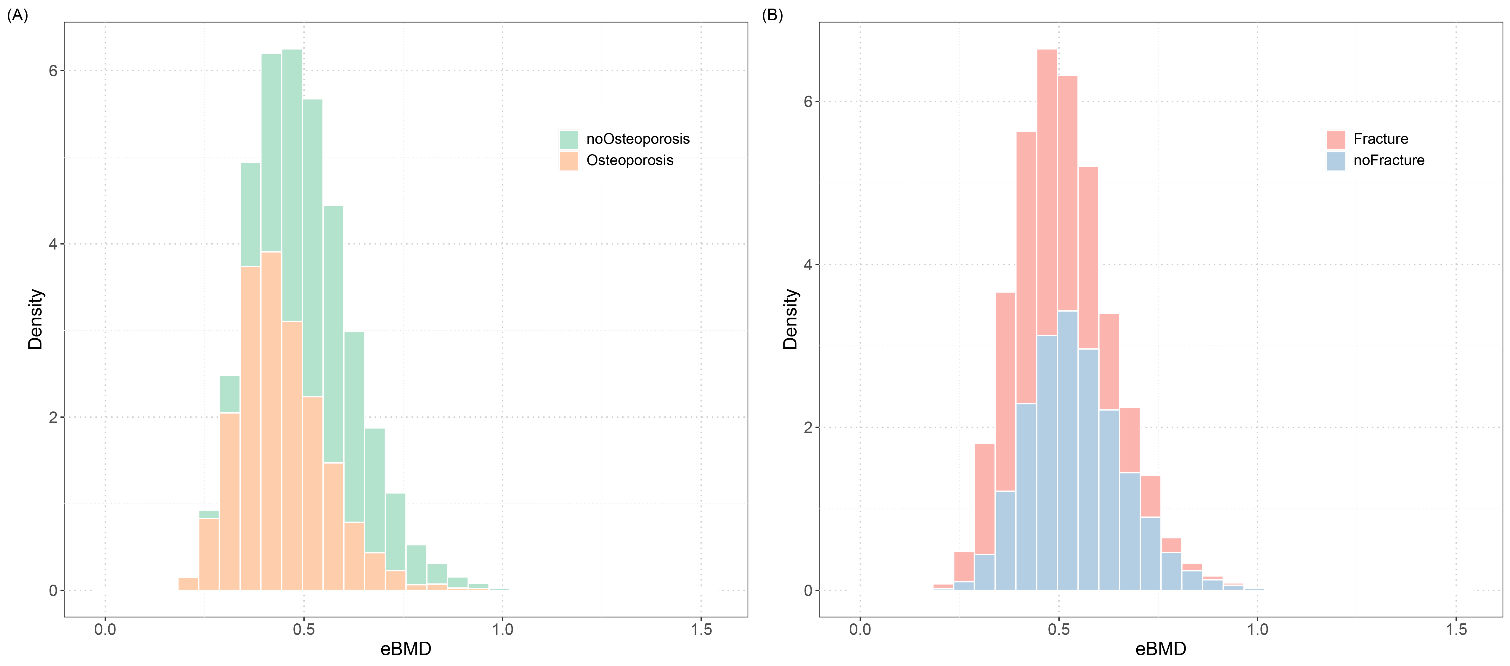
**Figure S1** (A) Frequency density plot of estimated bone mineral density in osteoporotic and non-osteoporotic individuals (Osteoporosis and Fracture,China,2022). (B) Frequency density plot of estimated bone mineral density in fracture and non-fracture individuals (Osteoporosis and Fracture,China,2022).

**
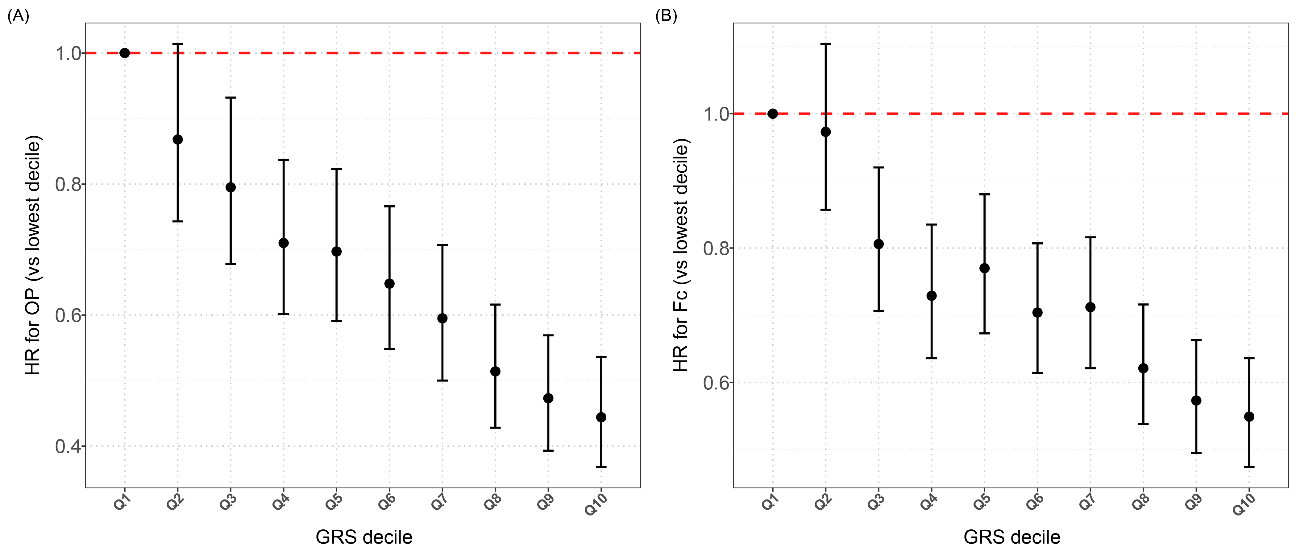
**

**Figure S2** (A) Decile analysis of genetic risk score on Osteoporosis risk between the participants with and without Osteoporosis in the test set (Osteoporosis and Fracture,China,2022). (B) Decile analysis of genetic risk score on fracture risk between the participants with and without fracture in the test set (Osteoporosis and Fracture,China,2022).


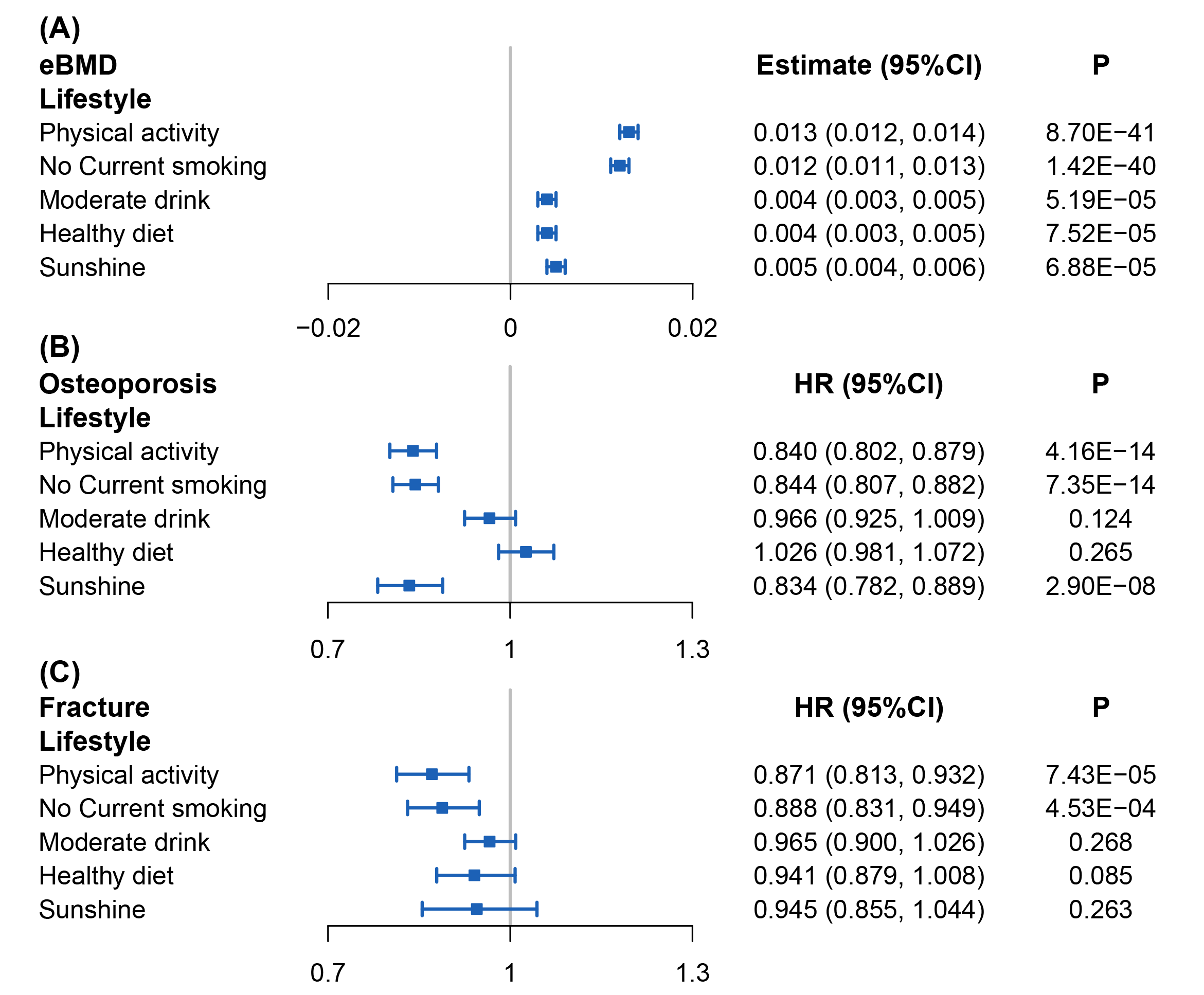
**Figre S3** (A)Association between 5 lifestyles and estimated bone mineral density in linear model (Osteoporosis and Fracture,China,2022). (B) Association between different 5 lifestyles and risk of incident osteoporosis in all participants prospective analysis (Osteoporosis and Fracture,China,2022). (C) Association between different 5 lifestyles and risk of incident fracture in all participants prospective analysis (Osteoporosis and Fracture,China,2022


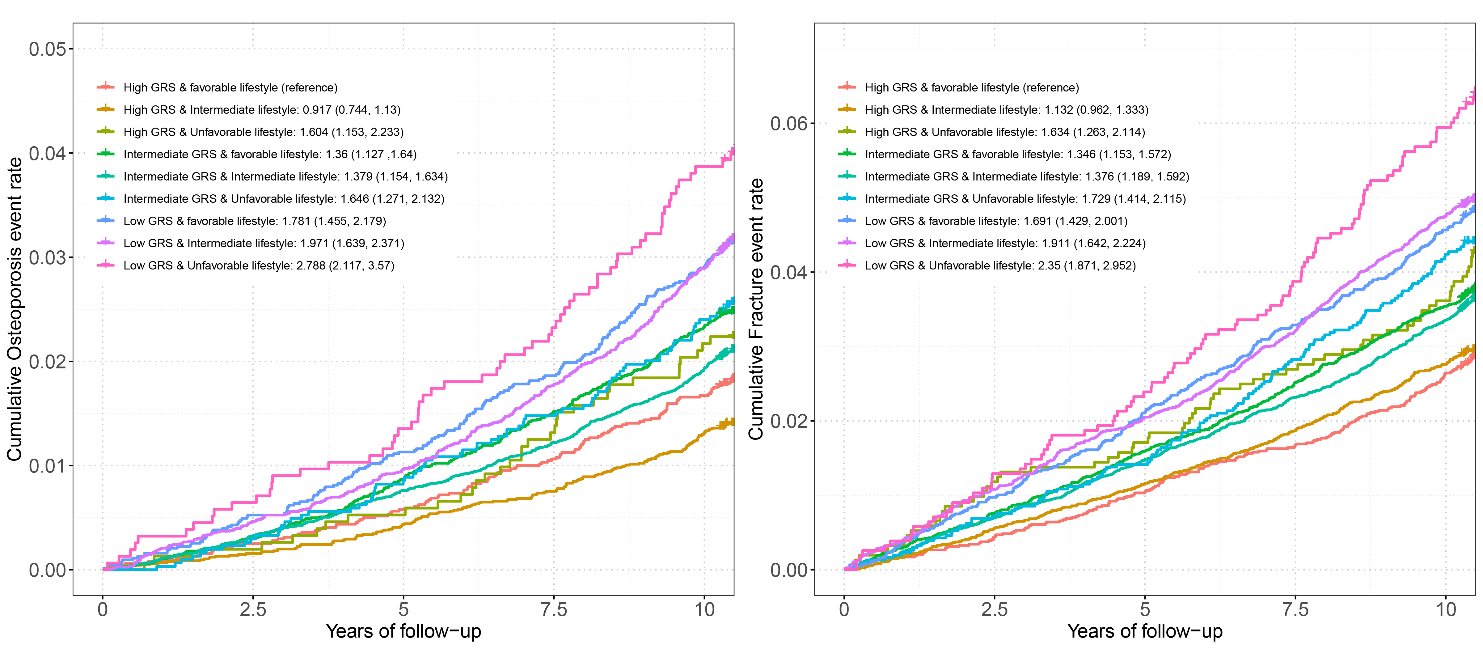


**Figure S4** Standardized rates of Osteoporosis /Fracture events in participants with different genetic risk score groups and different lifestyle groups in the UK Biobank cohort (Osteoporosis and Fracture,China,2022).

**Table S1**.Overview of the used International Classification of Diseases diagnosis

| **Diseases** | **Diagnostic classification codes**  **ICD10 codes (41720)**  *(Participants with at least one of the following diagnoses according to the electronic health registries)* |
| --- | --- |
| Osteoporosis | M80.0-M80.9, M81.0-M81.9, M82.0-M82.8 |
| Fracture | M48.4, M48.5, M80.0- M80.8, M84.3-M.84.4, S12.0-S12.9, S22.0-S22.9, S32.0-S32.8, S42.0-S42.9, S52.0-S52.9, S72.0-S72.9, S82.0-S82.9, Z87310, Z87311 |

codes to define cases (Osteoporosis and Fracture,China,2022).

Note: ICD = International Classification of Diseases

**Table S2.** Definition of healthy lifestyle factor (Osteoporosis and Fracture,China,2022).

| **Healthy lifestyle factor** | **Source and definition** | **UK Biobank field codes** |
| --- | --- | --- |
| No current smoking | Former smokers (smoking status) and who have quit smoking for more than 30 years are defined as no current smokers. | 20116, 2897 |
| Physical exercise | Regular physical exercise is defined as ≥ 150 minutes of moderate intensity activity per week, ≥ 75 minutes of vigorous exercise per week, at least 5 days of moderate intensity exercise per week and one vigorous exercise session per week | 884, 894, 904, 914 |
| Healthy diet | A healthy diet is defined as having five of the seven food groups, drinking milk (including all types of milk), eating ≥4 servings of fruit per day(Count one apple, one banana, 10 grapes etc as one piece; Count one prune, one dried apricot, 10 raisins as one piece), ≥4 servings of vegetables per day(Eating one heaped table spoon of cooked vegetables or salad or raw vegetables per day as one piece), ≥3 servings of whole grains(Eating 1 bowl of cereal per week as one piece), ≥2 servings of fish( Eating fish more than once a week), ≤1 serving of processed meat and ≤1.5 servings of unprocessed red meat per week | 1418, 1309, 1319, 1289, 1299, 1329, 1339, 1349, 1369, 1379, 1389, 1458, 1468 |
| Non-excessive alcohol consumption | " Non-excessive alcohol consumption " was defined as those who drank once or twice a week or one to three times a month or special occasions only. | 1558 |
| Sunshine | Sunshine is defined as the people who spend ≥ 2 hours outdoor in summer and spend ≥ 1 hours outdoor in winter. | 1050, 1060 |

**Table S3.** Associations between different Genetic risk scores and Fracture/ Osteoporosis risks (Osteoporosis and Fracture,China,2022).

| **Cut-off** | | **N*_SNP_*** | **eBMD** | | **Test set (Osteoporosis)** | | **Test set (Fracture)** | |
| --- | --- | --- | --- | --- | --- | --- | --- | --- |
|  |  |  | **OR (95%CI)** | ***P*** | **HR (95%CI)** | ***P*** | **HR (95%CI)** | ***P*** |
|  | **5.0E-05** | **1686** | **1.037(1.037,1.039)** | **2.01E-318** | **0.161(0.120,0.216)** | **5.13E-34** | **0.254(0.201,0.321)** | **3.85E-30** |
|  | 5.0E-06 | 1118 | 1.035(1.035,1.037) | 6.51E-277 | 0.163(0.119,0.223) | 4.85E-30 | 0.366(0.317,0.422) | 5.60E-27 |
|  | 5.0E-07 | 831 | 1.033(1.033,1.035) | 1.67E-246 | 0.160(0.115,0.222) | 4.11E-28 | 0.357(0.307,0.414) | 8.17E-27 |
|  | 5.0E-08 | 647 | 1.032(1.031,1.033) | 1.82E-226 | 0.157(0.111,0.221) | 3.20E-26 | 0.352(0.301,0.412) | 1.32E-25 |

Note: Associations were adjusted for age, sex, genotyped batch, Townsend deprivation index and the first 10 principal components of

ancestry. Abbreviations: HR, hazard ratio; CI, confidence interval; GRS, Genetic risk score.

**Table S4** Associations between Genetic risk score and Osteoporosis/Fracture in different sex-stratified groups in test set (Osteoporosis and Fracture,China,2022).

| **Subgroups** | **GRS** | **Osteoporosis** | | | **Fracture** | | |
| --- | --- | --- | --- | --- | --- | --- | --- |
|  |  | **Incidence/**  **100,000 py** | **HR(95%CI)** | ***P*** | **Incidence/**  **100,000 py** | **HR(95%CI)** | ***P*** |
| All | Low | 347.10 | **Ref** |  | 533.72 | **Ref** |  |
| All | Intermediate | 253.09 | 0.710(0.648,0.777) | 1.62E-13 | 399.14 | 0.744(0.691,0.801) | 4.08E-15 |
| All | High | 185.05 | 0.508(0.451,0.572) | 8.48E-29 | 185.04 | 0.601(0.548,0.66) | 4.81E-27 |
| Men | Low | 124.95 | **Ref** |  | 383.22 | **Ref** |  |
| Men | Intermediate | 100.02 | 0.854(0.681,1.072) | 0.173 | 303.66 | 0.801(0.705,0.91) | 6.44E-04 |
| Men | High | 75.52 | 0.641(0.48,0.855) | 2.49E-03 | 240.92 | 0.648(0.555,0.763) | 1.18E-07 |
| Women | Low | 540.73 | **Ref** |  | 664.32 | **Ref** |  |
| Women | Intermediate | 386.34 | 0.683(0.619,0.755) | 6.85E-14 | 481.75 | 0.717(0.655,0.785) | 5.19E-13 |
| Women | High | 277.99 | 0.484(0.425,0.552) | 2.32E-27 | 387.44 | 0.578(0.516,0.648) | 4.18E-21 |

Note: Defined by quartiles of GRS: low (the bottom quartiles), intermediate (quartiles 2-3) and high (the top quartiles). Associations were adjusted for age, sex, genotyped batch, Townsend deprivation index and the first 10 principal components of ancestry. HR, hazard ration; CI, confidence interval; GRS, Genetic risk score.

| **Gender** | **Lifestyle** | **Osteoporosis** | | | **Fracture** | | | |
| --- | --- | --- | --- | --- | --- | --- | --- | --- |
|  |  | **Incidence/**  **100,000 py** | **HR(95%CI)** | ***P*** | | **Incidence/**  **100,000 py** | **HR(95%CI)** | ***P*** |
| All | Favorable | 268.46 | **Ref** |  | | 389.74 | **Ref** |  |
| All | Intermediate | 237.59 | 1.03(0.943,1.125) | 0.514 | | 399.48 | 1.079(1.005,1.16) | 3.74E-02 |
| All | Unfavorable | 302.44 | 1.397(1.204,1.62) | 9.96E-06 | | 500.42 | 1.385(1.23,1.56) | 8.49E-08 |
| Men | Favorable | 70.31 | **Ref** |  | | 281.22 | **Ref** |  |
| Men | Intermediate | 94.66 | 1.176(0.92,1.503) | 0.196 | | 297.69 | 1.029(0.904,1.172) | 0.664 |
| Men | Unfavorable | 160.88 | 1.91(1.369,2.666) | 1.40E-04 | | 370.81 | 1.261(1.033,1.540) | 2.27E-02 |
| Women | Favorable | 389.378 | **Ref** |  | | 457.33 | **Ref** |  |
| Women | Intermediate | 378.66 | 1.014(0.922,1.115) | 0.781 | | 499.49 | 1.112(1.020,1.212) | 1.59E-02 |
| Women | Unfavorable | 458.32 | 1.293(1.087,1.536) | 3.62E-03 | | 643.00 | 1.487(1.281,1.726) | 1.83E-07 |

**Table S5.** Associations between healthy lifestyles and Osteoporosis/Fracture risks in different sex-stratified groups in test set (Osteoporosis and Fracture,China,2022).

Note: Participants were divided into favorable (4-5 healthy lifestyle factors), intermediate (2-3 healthy lifestyle factors), or unfavorable (0 or 1 healthy lifestyle factor) according to the number of lifestyle factors. Associations were adjusted for age, sex, genotyped batch, Townsend deprivation index and the first 10 principal components of ancestry.; HR, hazard ration; CI, confidence interval; GRS, Genetic risk score; Ref, reference.

**Table S6 Association between Genetic risk score and different lifestyles in test set (Osteoporosis and Fracture,China,2022).**

|  | **Beta** | **SD** | **T value** | **P** |
| --- | --- | --- | --- | --- |
| Lifestyle | 1.04E-03 | 8.40E-04 | 1.244 | 0.214 |
| Physical exercise | 8.50E-04 | 1.06E-03 | 0.804 | 0.421 |
| No current smoking | -7.52E-04 | 9.91E-04 | -0.759 | 0.448 |
| Moderate drink | 4.27E-04 | 9.65E-04 | 0.443 | 0.658 |
| Healthy diet | 1.38E-03 | 1.04E-03 | 1.319 | 0.187 |
| Sunshine | 2.27E-03 | 1.44E-03 | 1.580 | 0.114 |

Note: Lifestyle includes 5 types, Physical exercise, No current smoking, Moderate drink, Healthy diet and Sunshine respectively. Adjusted for age, sex, genotyped batch, assessment center, Townsend deprivation index and the first 10 principal components of ancestry.
